# Association between pre‐diagnostic serum albumin and cancer risk: Results from a prospective population‐based study

**DOI:** 10.1002/cam4.3937

**Published:** 2021-05-26

**Authors:** Zhuoyu Yang, Yadi Zheng, Zheng Wu, Yan Wen, Gang Wang, Shuohua Chen, Fengwei Tan, Jiang Li, Shouling Wu, Min Dai, Ni Li, Jie He

**Affiliations:** ^1^ Office of Cancer Screening National Cancer Center/National Clinical Research Center for Cancer/Cancer Hospital Chinese Academy of Medical Sciences and Peking Union Medical College Beijing China; ^2^ Department of Oncology Kailuan General Hospital Tangshan China; ^3^ Department of Health Care Center Kailuan General Hospital Tangshan China; ^4^ Department of Thoracic surgery National Cancer Center/National Clinical Research Center for Cancer/Cancer Hospital Chinese Academy of Medical Sciences and Peking Union Medical College Beijing China; ^5^ Chinese Academy of Medical Sciences Key Laboratory for National Cancer Big Data Analysis and Implement Beijing China; ^6^ Jiangsu Key Lab of Cancer Biomarkers, Prevention and Treatment Jiangsu Collaborative Innovation Center for Cancer Personalized Medicine Nanjing Medical University Nanjing China

**Keywords:** associations, cancer, Chinese, prospective study, serum albumin

## Abstract

**Background:**

Albumin is supposed to be associated with cancer risk. However, evidence on serum albumin and cancer risk among the Chinese population is sparse. This study was conducted to evaluate the association between pre‐diagnostic serum albumin and cancer risk among Chinese.

**Methods:**

A total of 82,061 participants with baseline information on serum albumin concentration in the Kailuan cohort were recruited. Cox proportional hazards models and restricted cubic spline (RCS) analyses were used to evaluate the association between pre‐diagnostic serum albumin and cancer risk.

**Results:**

Albumin levels were inversely associated with overall cancer risk (HR [95% CI]: Q2, Q3, Q4 vs. Q1: 0.91 [0.78–1.07], 0.80 [0.70–0.92], 0.73 [0.63–0.85]), and the risk of lung, colorectal, and liver cancer (HR [95% CI]: Q4 vs. Q1: lung: 0.70 [0.52–0.95], colorectal: 0.43 [0.26–0.72], liver: 0.59 [0.36–0.95]). After excluding new cancer cases within 2 years since enrollment, a more significant association was observed for liver cancer (HR [95% CI]: Q4 vs. Q1: 0.41 [0.21–0.78]), while associations converted to nonsignificant for lung and colorectal cancer. The RCS model suggested an inverse linear association between albumin and the risk of overall cancer (*p*‐overall < 0.0001, *p*‐nonlinear = 0.3716) and liver cancer (*p*‐overall = 0.0002, *p*‐nonlinear = 0.1807).

**Conclusions:**

Our findings suggest that pre‐diagnostic serum albumin is inversely and linearly associated with cancer risk among the Chinese population. This study provides evidence that albumin may be valuable to the prediction and stratification of cancer risk in the general population. However, the biological mechanism and clinical significance remain to be elucidated. Population studies with longer follow‐up time as well as experimental studies are further required.

## Graphical abstract

This is the first prospective cohort study to evaluate the association between pre‐diagnostic serum albumin and cancer risk. Inverse and linear associations were firstly reported between pre‐diagnostic serum albumin and the risks of overall cancer and liver cancer. These findings provide further evidence that albumin may be valuable to the prediction and stratification of cancer risk in the general population.

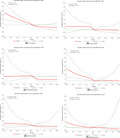

## 1 INTRODUCTION

With increasing incidence and mortality, cancer is now responsible for most global deaths in the 21st century. According to the report of the GLOBOCAN, an estimated 18.1 million new cancer cases and 9.6 million cancer deaths occurred worldwide in 2018, among which more than one fifth were from China.^1^

As cancer becoming a major public health problem, several important risk factors have been identified in the literature, such as smoking, drinking, obesity, and hepatitis B virus (HBV) infection,^2^, ^3^, ^4^, ^5^ with some generic to all cancers and some specific for different cancer types. Albumin was proved to be closely associated with the advance and prognosis of cancer since 1989.^6^, ^7^, ^8^, ^9^, ^10^, ^11^, ^12^, ^13^, ^14^, ^15^, ^16^ Hypotheses for decreased albumin concentration in cancer patients were varied, including malnutrition, increased albumin consumption due to the expression of cancer cells, and inhibition of albumin synthesis caused by systemic inflammation.^7^, ^17^ Furthermore, it was proposed that serum albumin was a kind of endogenous antioxidant,^18^ which could reduce cancer risk through exerting anticarcinogenic properties.^19^ A positive association between hypoalbuminemia and abnormal carcinoembryonic antigen were also reported.^20^ Taken together, albumin was supposed to be associated with cancer risk.

Previous studies on serum albumin levels and cancer risk have yielded inconsistent results.^21^, ^22^, ^23^, ^24^, ^25^, ^26^, ^27^, ^28^, ^29^, ^30^ Results from a cohort study indicated that lower albumin levels were associated with an increased risk of new cancer diagnosis within a year.^21^ Concerning each cancer type, inverse associations were confirmed by several studies between albumin levels and risks of colorectal^22^, ^23^ and breast cancer.^24^ However, there were also studies reporting nonsignificant associations among colorectal, breast, lung, and prostate cancer,^25^, ^26^, ^27^, ^28^, ^29^ and even positive associations among ovarian cancer ^30^ were reported.

Notably, among the above‐mentioned studies, only a few were prospective, and none was conducted among the Chinese population. Therefore, we conducted this study using data from the Kailuan cohort to confirm and further evaluate the association between pre‐diagnostic serum albumin and cancer risk among Chinese.

## 2 MATERIALS AND METHODS

### 2.1 Study population

The data were obtained from the Kailuan cohort study, which was a prospective study based on health examinations among employees of the Kailuan Company. The Kailuan Company is in Tangshan City, Hebei Province, and has developed a diversified economy, including coal mining, electric power, manufacturing, transportation, trading, and health care. Routine medical examinations of the employees were conducted every 2 years at 11 affiliated hospitals of the Kailuan Company. The Kailuan cohort study was initiated in May 2006. Four rounds of questionnaire interviews and health examinations were conducted in 2006–2007, 2008–2009, 2010–2011, and 2012–2013 to collect information on both risk factors and blood tests. New participants beyond the first round could enter the cohort at the second, third, and fourth round. Therefore, the Kailuan cohort was established as a population‐based dynamic cohort. In case of the potential survival bias, subjects with a previous diagnosis of any prevalent cancers before baseline recruitment were excluded. In total, 1,38,150 subjects were enrolled through four rounds of surveys and examinations, among which, participants without blood test results of serum albumin were further excluded for this analysis. Ultimately, 82,061 participants in the age range between 18 and 104 were enrolled in this study. The study was approved by the Medical Ethics Committee of Kailuan General Hospital and all participants provided written informed consent.

### 2.2 Laboratory tests

Standard protocols were applied for all measurements as described in previous studies.^31^, ^32^, ^33^ Morning fasting venous blood samples were collected from the antecubital vein and transfused into vacuum tubes containing ethylenediaminetetraacetic acid (EDTA). Tubes were centrifuged at 3000 g for 10 min at room temperature and blood samples were processed into plasma, serum, and other samples. Serum samples were detected within 4 hours. As for serum albumin, it was measured using an autoanalyzer (Hitachi 747; Hitachi) according to the manufacturer's protocols at the central laboratory of the Kailuan General Hospital. Four categories of baseline serum albumin levels were classified according to quartiles of the study population (Q1: <44.6, Q2: 44.6–45.9, Q3: 46.0–47.9, Q4: ≥48.0 g/L).

### 2.3 Assessment of covariables

Face‐to‐face interviews were conducted by well‐trained staff using standardized questionnaires to collect information on demographics, socioeconomics, lifestyle factors, and personal medical history as potential risk factors. The status of coal mine dust exposure was obtained from the participants' work history recorded in the Kailuan Company.^34^, ^35^ In the questionnaire, family income per person was classified as <500 Chinese Yuan (CNY) per month, 500–1000 CNY per month, and ≥1,000 CNY per month, referred to the low‐income standard of Gross National Income (less than $1005) by the World Bank. Educational level was divided into four categories including illiterate or primary school, junior high school, senior high school, and college and above. Smoking status was classified as never, quit, occasional (<1 cigarette per day), and frequent smoking (≥1 cigarette per day). Alcohol drinking status was divided into ever drinkers or never drinkers. Coal dust exposure status was classified as non‐exposure or exposure. Bodyweight and height were measured with participants wearing light clothes and no shoes, and the body mass index (BMI) was calculated according to the equation that BMI = weight (kg)/height (m^2^). Enzyme‐linked immunosorbent assay was used to detect HBV infection status and the status was classified as HBsAg positive or negative. Serum C‐reactive protein (CRP) was measured using a commercial, high‐sensitivity nephelometry assay (Cias Latex CRP‐H, Kanto Chemical Co. Inc.).

### 2.4 Outcome ascertainment

Follow‐up of each participant began when baseline examinations were completed and ended at the first diagnosis of cancer, death, last documented follow‐up contact, or 31 December 2015, whichever came first. Incident cancer cases were identified mainly through biennial interviews. Medical records and death certificates were also used to add missing outcome information, which were attained from the medical insurance system of Tangshan city, the social security system of the Kailuan Group, and discharge summaries of affiliated hospitals. The diagnoses of incident cancers were confirmed by clinical experts according to comprehensive information of pathological, imaging diagnosis, and blood biochemical examination. Cancers were coded according to the International Classification of Diseases, Tenth Revision (ICD–10). Gastric, colorectal, liver, lung, and breast cancer were coded as C16, C18–19, C22, C34, and C50, respectively.

### 2.5 Statistical analyses

Baseline characteristics of the participants were described by percentages and compared using the Chi‐square test for categorical variables. For continuous variables, distributions were described by the mean (standard deviation) and compared using analysis of Variance. To evaluate the association between baseline serum albumin levels and incident cancer risk, cox proportional hazards regression models were used to calculate hazard ratios (HRs) and 95% confidence intervals (CIs). Multivariable analyses were adjusted for potential confounders, including age (10‐year interval), gender (male, female), the education level (illiterate or primary school, junior high school, senior high school, and college and above), income status (<500 CNY, 500–1000 CNY, and ≥1000 CNY, per month), coal dust exposure status (non‐exposure or exposure), BMI (BMI < 18.5, 18.5 ≤ BMI < 25.0, 25.0 ≤ BMI < 30.0, BMI ≥ 30.0), the concentration of CRP (<0.5, 0.5–1.0, 1.1–2.5, >2.5, mg/L), smoking status (never, quit, <1 cigarette per day, ≥1 cigarette per day), alcohol drinking status (never, ever) and HBV infection status (HBsAg negative, HBsAg positive). Further subgroup analyses were conducted by subgroups of gender, BMI, and cigarette smoking status. For sensitivity analysis, we further excluded 563 participants who were diagnosed with cancers within the first 2 years after finishing the baseline examination of serum albumin. Moreover, the association between albumin concentrations and cancer risk were evaluated through restricted cubic spline (RCS) analysis based on multivariate Cox proportional hazard models.^36^ Tests in this study were all two‐sided and *p* < 0.05 was considered statistically significant. All statistical analyses were conducted using the SAS statistical software, version 9.4 (SAS Institute Inc.).

## 3 RESULTS

### 3.1 Baseline characteristics of participants

Selected baseline characteristics of participants in the Kailuan cohort were listed in Table 1. This study consisted of 62,141 males and 19,920 females with a mean age of 50.32 ± 12.81 at baseline. More than half of the participants have been exposed to coal dust or were supposed to be overweight according to the WHO standard. The rate of current smoking and ever alcohol drinking were 32.04% and 40.58%, respectively, and the prevalence of HBV infection status was 2.96% in this study. Among the study population, mean serum concentrations of albumin were 46.47±7.90 g/L. Lower serum albumin levels were mainly observed among individuals above 60 years old (proportion of Q1: 35.30), individuals with education levels below primary school (proportion of Q1: 33.38) and those with income less than 500 CNY (proportion of Q1: 27.42).

**TABLE 1:** Baseline characteristics of participants in the Kailuan cohort by serum albumin level

Characteristics	Serum albumin (g/L)						*χ* ^2^	*p*‐value
		Quartile 1	Quartile 2	Quartile 3	Quartile 4	Total		
	Mean ± SD	No. (%^a^)	No. (%^a^)	No. (%^a^)	No. (%^a^)	No. (%^b^)		
Total	46.47 ± 7.90	<44.6	44.6–45.9	46.0–47.9	≥48.0			
Gender								
Female	45.88 ± 8.07	5822 (29.23)	3998 (20.07)	6079 (30.52)	4021 (20.19)	19920 (24.27)	904.9050	<0.0001
Male	46.66 ± 7.84	14296 (23.01)	10473 (16.85)	18412 (29.63)	18960 (30.51)	62141 (75.73)		
Age (year)								
<40	47.54 ± 7.30	2289 (14.73)	1959 (12.60)	4587 (29.51)	6709 (43.16)	15544 (18.94)	3721.2277	<0.0001
40–49	46.53 ± 6.94	4732 (22.52)	3800 (18.08)	6541 (31.13)	5939 (28.26)	21012 (25.61)		
50–59	46.34 ± 7.62	6970 (24.76)	5410 (19.22)	8640 (30.28)	7127 (25.32)	28147 (34.30)		
≥60	45.66 ± 9.66	6127 (35.30)	3302 (19.02)	4723 (27.21)	3206 (18.47)	17358 (21.15)		
Income (yuan/month)								
<500	46.09 ± 6.01	4448 (27.42)	2924 (18.02)	4870 (30.02)	3980 (24.53)	16222 (20.80)	825.3477	<0.0001
500–1000	46.43 ± 8.91	10448 (24.20)	8225 (19.05)	13252 (30.70)	11246 (26.05)	43171 (55.36)		
≥1000	46.95 ± 7.01	4259 (22.90)	2665 (14.33)	5027 (27.03)	6644 (35.73)	18595 (23.84)		
Education								
Illiterate or primary school	45.80 ± 7.72	2251 (33.38)	1194 (17.70)	1823 (27.03)	1476 (21.89)	6744 (8.54)	824.3316	<0.0001
Junior high school	46.44 ± 7.64	12590 (24.06)	9860 (18.84)	15887 (30.35)	14001 (26.75)	52338 (66.31)		
Senior high school	46.97 ± 6.37	2663 (22.86)	1687 (14.48)	3208 (27.53)	4093 (35.13)	11651 (14.76)		
College and above	46.72 ± 11.93	1881 (22.94)	1185 (14.45)	2480 (30.24)	2655 (32.37)	8201 (10.39)		
Smoking status								
Never	46.38 ± 8.49	12263 (25.57)	8817 (18.38)	14205 (29.62)	12679 (26.43)	47964 (64.91)	273.0513	<0.0001
Quit	46.48 ± 9.11	549 (24.34)	361 (16.00)	699 (30.98)	647 (28.68)	2256 (3.05)		
Occasional (<1 cigarette per day)	47.26 ± 10.33	330 (19.86)	264 (15.88)	467 (28.10)	601 (36.16)	1662 (2.25)		
Frequent (≥1 cigarette per day)	46.68 ± 6.58	5016 (22.79)	3559 (16.17)	6583 (29.90)	6856 (31.14)	22014 (29.79)		
Alcohol drinking status								
Never drinker	46.35 ± 8.34	12170 (25.87)	8767 (18.64)	13867 (29.48)	12235 (26.01)	47039 (59.42)	339.0104	<0.0001
Ever drinker	46.70 ± 7.56	7278 (22.65)	5196 (16.17)	9591 (29.85)	10066 (31.33)	32131 (40.58)		
Coal dust exposure status								
Non‐exposure	46.46 ± 9.28	8650 (25.84)	5813 (17.36)	9726 (29.05)	9288 (27.74)	33477 (42.46)	50.9545	<0.0001
Exposure	46.51 ± 6.99	10720 (23.63)	8102 (17.86)	13650 (30.09)	12890 (28.42)	45362 (57.54)		
BMI (kg/m^2^)								
BMI < 18.5	46.48 ± 3.09	490 (23.91)	324 (15.81)	595 (29.04)	640 (31.23)	2049 (2.51)	78.7321	<0.0001
18.5 ≤ BMI < 25.0	46.38 ± 8.49	9810 (25.41)	6948 (18.00)	11456 (29.68)	10388 (26.91)	38602 (47.31)		
25.0 ≤ BMI < 30.0	46.55 ± 7.39	7481 (23.83)	5458 (17.39)	9442 (30.08)	9010 (28.70)	31391 (38.47)		
BMI ≥ 30.0	46.62 ± 7.92	2180 (22.82)	1676 (17.55)	2859 (29.93)	2837 (29.70)	9552 (11.71)		
HBsAg status								
Negative	46.50 ± 7.95	18205 (24.30)	13293 (17.75)	22345 (29.83)	21062 (28.12)	74905 (97.04)	39.7021	<0.0001
Positive	46.06 ± 3.45	678 (29.72)	346 (15.17)	623 (27.31)	634 (27.79)	2281 (2.96)		
Crp	N.A.	2.95 ± 9.21	2.63 ± 6.31	2.48 ± 5.21	2.55 ± 6.05	2.64 ± 6.79	108.6861	<0.0001

Note

For Crp, mean ± SD were calculated in each quartile, and *t* value and relevant *p* value were provided.

Abbreviation: BMI, body mass index; Crp, C‐reactive protein.

^a^ Row percentage.

^b^ Column percentage.

### 3.2 Risk associations

Results of Cox regression analyses on serum albumin levels and cancer risk were shown in Table 2. Till 31 December 2015, a total of 1482 new cancer cases occurred with a median follow‐up duration of 4.69 years. After multivariable adjustment for potential confounders (age, gender, education level, income, coal dust exposure, concentration of CRP, BMI, frequency of smoking, frequency of alcohol drinking, and HBsAg infection status), participants with higher serum albumin levels were at decreased cancer risk of 9%–27%, compared with those whose serum albumin concentrations were below 44.6 g/L (Q2 vs. Q1: HR 0.91, 95% CI 0.78–1.07; Q3 vs. Q1: HR 0.80, 95% CI 0.70–0.92; Q4 vs. Q1: HR 0.73, 95% CI 0.63–0.85). With respect to cancers of specific types, albumin levels were significantly inversely associated with risks of lung cancer (Q2 vs. Q1: HR 0.82, 95% CI 0.60–1.12; Q3 vs. Q1: HR 0.68, 95% CI 0.51–0.90; Q4 vs. Q1: HR 0.70, 95% CI 0.52–0.95), colorectal cancer (Q2 vs. Q1: HR 0.87, 95% CI 0.57–1.34; Q3 vs. Q1: HR 0.95, 95% CI 0.65–1.38; Q4 vs. Q1: HR 0.43, 95% CI 0.26–0.72), and liver cancer (Q2 vs. Q1: HR 0.55, 95% CI 0.31–0.95; Q3 vs. Q1: HR 0.44, 95% CI 0.26–0.73; Q4 vs. Q1: HR 0.59, 95% CI 0.36–0.95). In the sensitivity analysis, higher albumin levels remained to be associated with decreased overall cancer risks (Q2 vs. Q1: HR 0.93, 95% CI 0.76–1.13; Q3 vs. Q1: HR 0.83, 95% CI 0.69–0.99; Q4 vs. Q1: HR 0.75, 95% CI 0.61–0.91), and significantly lower risk of liver cancer was found related to higher albumin levels (Q2 vs. Q1: HR 0.49, 95% CI 0.25–0.97; Q3 vs. Q1: HR 0.40, 95% CI 0.22–0.74; Q4 vs. Q1: HR 0.41, 95% CI 0.21–0.78). However, no significant associations between albumin levels and risks of lung and colorectal cancer were observed after excluding participants who had new cancer diagnoses within 2 years since enrollment.

**TABLE 2:**
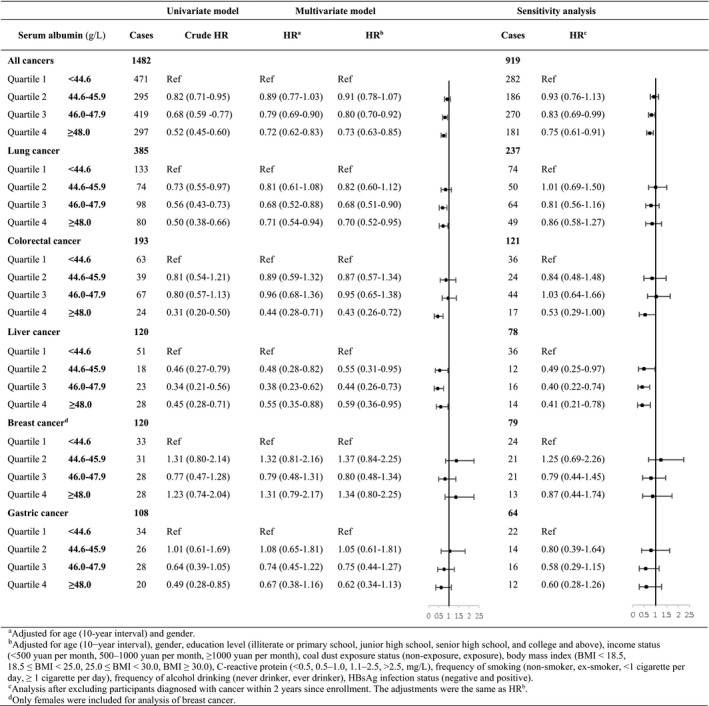
The association between serum albumin and cancer risk, Kailuan cohort

Subgroup analyses by gender, BMI, and smoking status were displayed in Table 3. The associations between albumin levels and cancer risks were not significant among females, while for males, inverse associations remained. In general, individuals with higher albumin levels were at decreased overall cancer risk among both the normal‐weight and overweight population. However, for lung cancer risk, a significant inverse association was only observed among normal weight individuals, while for risk of colorectal cancer, the inverse association was only significant among the overweight. Moreover, high levels of albumin were found to be associated with lung, colorectal, and liver cancer especially among non‐smokers.

**TABLE 3:** Hazard ratios (HRs) and 95% confidence intervals (CIs) of cancer risk in relationship to serum albumin by gender, body mass index (BMI), and smoking status, Kailuan cohort

Serum albumin (g/L)		Gender		BMI		Smoking status	
		Male	Female	Normal weight (BMI < 25 kg/m^2^)	Overweight (BMI ≥ 25 kg/m^2^)	Non‐smoker	Ever smoker
All cancers							
Quartile 1	**<44.6**	Ref	Ref	Ref	Ref	Ref	Ref
Quartile 2	**44.6–45.9**	0.86 (0.72–1.03)	1.08 (0.81–1.44)	0.89 (0.72–1.11)	0.94 (0.76–1.17)	0.93 (0.77–1.12)	0.89 (0.69–1.16)
Quartile 3	**46.0–47.9**	0.82 (0.70–0.97)	0.80 (0.61–1.06)	0.83 (0.68–1.01)	0.78 (0.64–0.96)	0.77 (0.65–0.92)	0.86 (0.69–1.08)
Quartile 4	**≥48.0**	0.73 (0.61–0.87)	0.85 (0.61 −1.18)	0.74 (0.59–0.92)	0.73 (0.58–0.91)	0.75 (0.61–0.91)	0.73 (0.56–0.93)
Lung cancer							
Quartile 1	**<44.6**	Ref	Ref	Ref	Ref	Ref	Ref
Quartile 2	**44.6–45.9**	0.76 (0.54–1.07)	1.18 (0.56–2.48)	0.72 (0.47–1.09)	0.97 (0.62–1.52)	0.82 (0.55–1.24)	0.81 (0.51–1.29)
Quartile 3	**46.0–47.9**	0.70 (0.52–0.95)	0.52 (0.22–1.24)	0.63 (0.43–0.93)	0.75 (0.49–1.14)	0.51 (0.34–0.77)	0.89 (0.60–1.32)
Quartile 4	**≥48.0**	0.69 (0.50–0.96)	0.85 (0.34–2.11)	0.60 (0.39–0.92)	0.84 (0.54–1.31)	0.71 (0.47–1.08)	0.71 (0.45–1.12)
Colorectal cancer							
Quartile 1	**<44.6**	Ref	Ref	Ref	Ref	Ref	Ref
Quartile 2	**44.6–45.9**	0.74 (0.45–1.21)	1.61 (0.62–4.18)	1.16 (0.62–2.18)	0.67 (0.37–1.23)	0.78 (0.45–1.33)	1.07 (0.52–2.21)
Quartile 3	**46.0–47.9**	0.86 (0.57–1.31)	1.36 (0.55–3.35)	1.28 (0.73–2.23)	0.73 (0.44–1.22)	0.94 (0.59–1.48)	0.97 (0.50–1.86)
Quartile 4	**≥48.0**	0.40 (0.23–0.69)	0.61 (0.16–2.32)	0.78 (0.39–1.53)	0.22 (0.10–0.51)	0.39 (0.20–0.76)	0.53 (0.23–1.21)
Breast cancer^a^							
Quartile 1	**<44.6**	N.A.	Ref	Ref	Ref	Ref	N.A.
Quartile 2	**44.6–45.9**	N.A.	1.37 (0.84–2.25)	2.04 (0.88–4.71)	1.14 (0.60–2.15)	1.42 (0.86–2.33)	N.A.
Quartile 3	**46.0–47.9**	N.A.	0.80 (0.48–1.34)	1.34 (0.58–3.07)	0.57 (0.28–1.16)	0.83 (0.49–1.39)	N.A.
Quartile 4	**≥48.0**	N.A.	1.34 (0.80–2.25)	1.91 (0.81–4.52)	1.13 (0.57–2.22)	1.39 (0.82–2.34)	N.A.
Liver cancer							
Quartile 1	**<44.6**	Ref	Ref	Ref	Ref	Ref	Ref
Quartile 2	**44.6–45.9**	0.47 (0.26–0.87)	1.71 (0.34–8.60)	0.45 (0.18–1.11)	0.63 (0.31–1.27)	0.64 (0.33–1.27)	0.40 (0.15–1.06)
Quartile 3	**46.0–47.9**	0.44 (0.26–0.75)	0.34 (0.04–3.25)	0.44 (0.20–0.97)	0.45 (0.23–0.87)	0.36 (0.18–0.75)	0.55 (0.27–1.13)
Quartile 4	**≥48.0**	0.58 (0.35–0.95)	0.73 (0.07–7.15)	0.53 (0.24–1.15)	0.63 (0.33–1.17)	0.52 (0.26–1.02)	0.68 (0.34–1.37)
Gastric cancer^b^							
Quartile 1	**<44.6**	Ref	N.A.	Ref	Ref	Ref	Ref
Quartile 2	**44.6–45.9**	1.20 (0.66–2.18)	N.A.	1.06 (0.50–2.23)	1.04 (0.47–2.30)	1.22 (0.62–2.40)	0.80 (0.32–2.01)
Quartile 3	**46.0–47.9**	0.91 (0.52–1.62)	N.A.	0.85 (0.42–1.71)	0.65 (0.29–1.44)	0.62 (0.30–1.29)	0.94 (0.44–2.02)
Quartile 4	**≥48.0**	0.78 (0.42–1.47)	N.A.	0.57 (0.24–1.34)	0.67 (0.29–1.58)	0.76 (0.35–1.63)	0.47 (0.18–1.24)

Note

Adjusted for age (10‐year interval), gender, education level (illiterate or primary school, junior high school, senior high school, and college and above), income status (<500 yuan per month, 500–1000 yuan per month, ≥1000 yuan per month), coal dust exposure status (non‐exposure, exposure), BMI (BMI <18.5, 18.5 ≤ BMI <25.0, 25.0 ≤ BMI <30.0, BMI ≥30.0), C‐reactive protein (<0.5, 0.5–1.0, 1.1–2.5, >2.5, mg/L), frequency of smoking (non‐smoker, ex‐smoker, <1 cigarette per day, ≥1 cigarette per day), frequency of alcohol drinking (never drinker, ever drinker), HBsAg infection status (negative and positive). Grouping variables were excluded for each subgroup.

^a^ Only females were included for analysis of breast cancer. For female ever smokers, the analysis was infeasible due to the limitation of subjects’ number.

^b^ The analysis of gastric cancer risk among female was infeasible due to the limitation of cancer cases.

**FIGURE 1:**
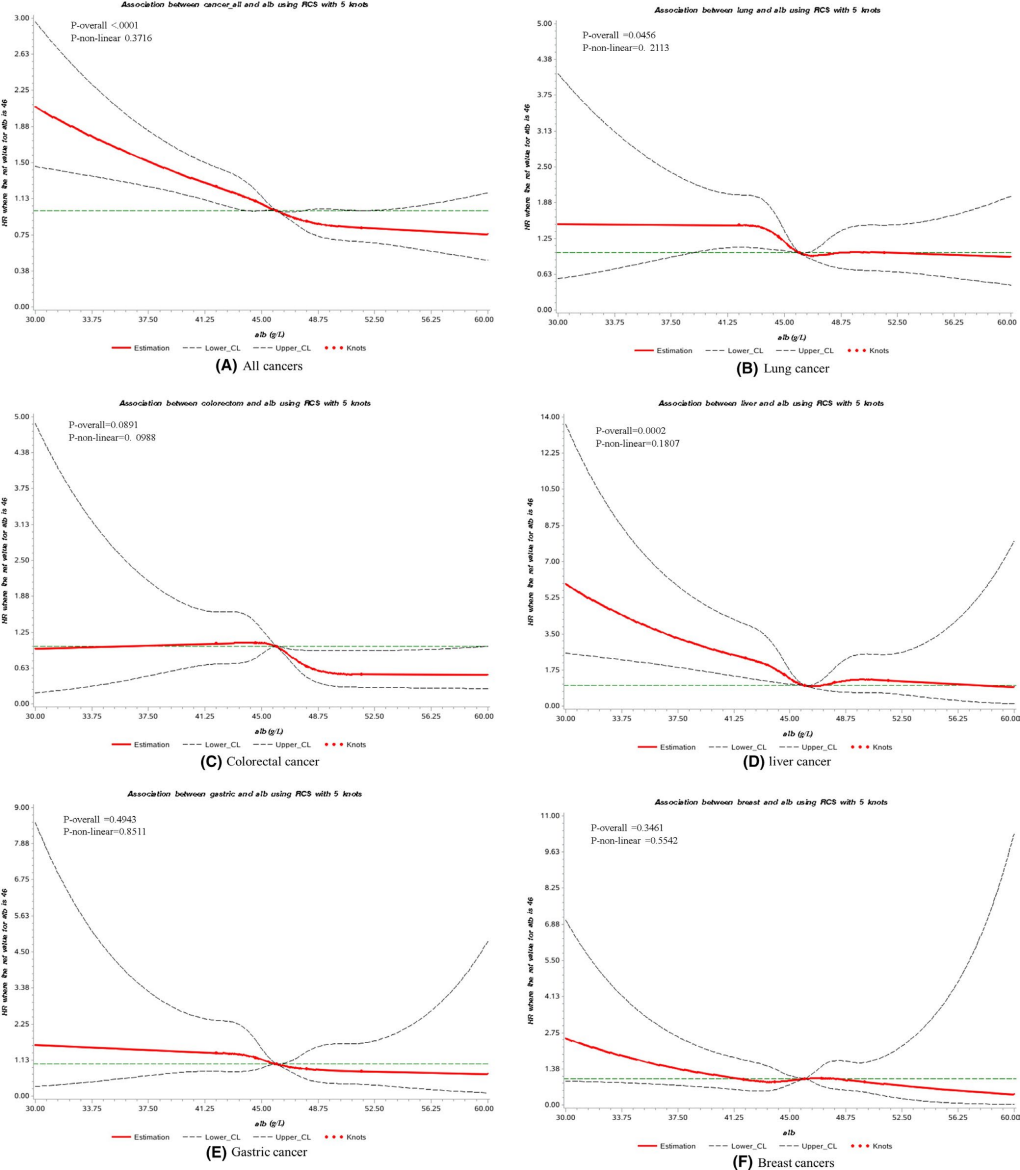
Cubic spline graph of the adjusted HR (represented by solid line) and 95% CI (represented by the dotted lines) for the association between serum albumin and risk of primary cancer in Kailuan cohort, 2006–2013. (A) All cancers, (B) lung cancer, (C) colorectal cancer, (D) liver cancer, (E) gastric cancer, (F) breast cancer. Knots: 42, 44.6,46, 48 and 51.5 (95th) of the distribution of serum albumin concentration (g/L); Reference: 46 g/L, 50th of the distribution of serum albumin concentration. Analyses are adjusted for age ^10−yearinterval^, gender, education level (illiterate or primary school, junior high school, senior high school, and college and above), income status (<500 yuan per month, 500–1000 yuan per month, ≥1000 yuan per month), coal dust exposure status (non‐exposure, exposure), BMI (BMI <18.5, 18.5 ≤ BMI <25.0, 25.0 ≤ BMI <30.0, BMI ≥30.0), C‐reactive protein (<0.5, 0.5–1.0, 1.1–2.5, >2.5, mg/​L), smoking status (never, quit, <1 cigarette per day, ≥1 cigarette per day), alcohol drinking status (never, ever) and HBV infection status (HBsAg negative, HBsAg positive). BMI, body mass index; HBV, hepatitis B virus

The RCS model showed a significant linear association between serum albumin concentrations and overall cancer risk among the subjects (*p*‐overall < 0.0001, *p*‐nonlinear = 0.3716). As the 50th quintile of albumin concentrations (46 g/L) was chosen to be the reference, the HRs of overall cancer risk related to albumin levels rise sharply when albumin levels were below 46 g/L. Concerning each cancer type, there was a linear association between albumin and liver cancer risk (*p*‐overall = 0.0002, *p*‐nonlinear = 0.1807), and a slightly significant association was also observed for lung cancer (*p*‐overall = 0.0456, *p*‐nonlinear = 0.2113) (Figure 1).

## 4 DISCUSSION

Based on a large prospective cohort study, this analysis displayed the associations between pre‐diagnostic serum albumin and cancer risks. After controlling for pertinent risk factors, results suggested that serum albumin was inversely associated with overall cancer risk with a significant linear association. When analyzed by cancer type, the association between albumin and overall cancer risk was mainly attributed to lung, colorectal and especially, liver cancer. Moreover, a significant inverse linear relationship was observed between serum albumin concentration and liver cancer risk.

Our findings suggested that pre‐diagnostic albumin levels were inversely associated with overall cancer risk. This inverse association was in line with a recent study based on a UK database of adult primary care patients.^21^ Additionally, we observed a significant linear relationship between serum albumin concentrations and cancer risk. Referred to the median of albumin concentrations, HRs of overall cancer risk increased sharply when albumin concentrations were below 46 g/L.

For decades, it has been confirmed that albumin was associated with the overall mortality,^6^, ^8^ and especially the prognosis of cancer,^7^, ^9^, ^10^, ^11^, ^12^, ^13^, ^14^, ^15^, ^16^ whereas only a few studies were conducted concerning cancer risk. Hypotheses for decreased albumin concentration with increased cancer risk raised by the previous study were varied from malnutrition, increased albumin consumption due to the expression of cancer cells, to antioxidant properties and inhibition of albumin synthesis caused by systemic inflammation. First, albumin is one of the various inflammation‐related markers. In previous studies, pooled analyses of inflammation‐related markers including the ratio of albumin to globulin,^37^ C‐reactive protein to albumin,^38^ albumin‐to‐alkaline phosphatase,^39^ and albumin to fibrinogen^40^ were conducted concerning cancer prognosis. For these indexes, the prognostic value of clinical significance has already been proved. Considering that serum albumin is a negative acute‐phase protein, inflammation could suppress albumin synthesis and lead to low albumin concentrations. A longitudinal study has observed decreased albumin in women with early‐stage ovarian cancer from a mean of 51.3–40.9 g/L (*p* < 0.001).^41^ Though few studies explained the mechanism of the influence of serum albumin on cancer risk and mortality, a previous study conducted among dialysis patients suggested that the influence of serum albumin on mortality was partly explained by the inflammatory pathway.^42^ Besides, albumin is supposed to be an important extracellular antioxidant.^18^ In animal experiments, serum albumin was shown to be protective through reducing arterial reactivity in endotoxemia as an antioxidant and improving the anti‐inflammatory effect. However, no further research explored the mechanism of albumin in relation to cancer in the human body. Two previous studies have explored the association between cancer risk, mortality, and metabolic markers including albumin, bilirubin, and uric acid, which were all considered as non‐nutrient antioxidants of physiologic importance. A nested case‐control study showed that the risk of colon cancer increased from the highest to the lowest quartiles of serum albumin (*p*_trend_ = 0.05) whereas no statistically significant dose‐response trend was observed for bilirubin or uric acid.^22^ Another cohort study also reported that albumin and uric acid levels were associated with breast cancer risk. The theory of extracellular antioxidants was still insufficient and required further exploration. Moreover, cancer is recognized as a kind of consumptive disease with clinical symptoms including weight loss in the late stages. It was supposed that a lower level of serum albumin associated with higher cancer risk may indicate the influence on nutrition intake before cancer diagnoses. However, there was no prior hypothesis concerning nutrition intake and cancer risk and this just remains as a supposition.

With respect to liver cancer risk, we observed an inverse association with albumin levels, and after excluding participants who were diagnosed with cancer within 2 years since enrollment, a more significant association was observed. Significant linear associations were also suggested by the RCS model for liver cancer. Though there was no previous research exploring the association between albumin and liver cancer risk, it has been confirmed that albumin was produced by hepatocytes and abnormal albumin levels were in relation to hepatic dysfunction.^43^, ^44^

For colorectal cancer, we observed an inverse association between pre‐diagnostic albumin levels and colorectal cancer risk, which was consistent with the results of several previous studies.^22^, ^23^, ^45^ Nevertheless, there were also two studies reporting nonsignificant associations between albumin and colon cancer risk.^25^, ^26^ Albumin was suggested to play a role in the resistance of the gastrointestinal mucosal membrane to lipid peroxidation.^46^ Therefore, it is believed that albumin is closely related to the development of gastrointestinal cancer and many studies focused on its association with risks of gastrointestinal cancer, especially colon cancer.^22^, ^23^, ^25^, ^26^ Furthermore, a previous study showed that hypoalbuminemia in patients with colorectal cancer reflects both increased nutritional risk and greater systemic inflammatory response.^47^

For lung cancer, the inverse association was observed between lung cancer risk and albumin concentrations. However, the inverse association converted to nonsignificant after excluding participants who had cancer diagnoses within 2 years since recruitment. Besides, there was also a cohort study reporting nonsignificant results between albumin and lung cancer.^29^ Though the physiological and functional properties of the lung have little to do with albumin, the association between albumin and lung cancer risk may reflect systemic inflammation. However, the inverse association between lung cancer risk and serum albumin was novel and should be interpreted cautiously.

In our analysis, nonsignificant associations were observed for cancer subtypes including gastric and breast cancer, which might be related to the limited number of cancer cases and low proportion of females in the study population. The lack of association between breast cancer risk and albumin in our study was consistent with findings of the AMORIS study,^27^ whereas a recent cohort study reported a weak inverse relationship,^24^ with the HRs being 0.71 (95% CI 0.51–0.99) for participants in the highest albumin quartile. However, there was no previous study reporting the association related to gastric cancer.

Taken together, the positive results in our analysis could only indicate the association between serum albumin and cancer risk. Lack of research on the basic biological mechanism, these results should be interpreted with caution and further evidence on biological mechanism was required.

Since gender, smoking status, and BMI were suggested to be closely associated with cancer risk,^2^ we further conducted subgroup analyses by these factors. Significant inverse associations were observed especially for the non‐smokers and males. Concerning lung cancer risk, an inverse association was observed among normal‐weight individuals, whereas for colorectal cancer, a significant association was observed among the overweight. As suggested in studies on BMI and cancer risk, obesity was suggested as a risk factor of colorectal cancer,^48^ whereas a protective effect was indicated for lung cancer.^5^ Taken together, our findings were consistent with the significant association between obesity and cancer risks. As the established risk factors played a leading role in the development of cancer, the effect of albumin was still significant.

The present study had several limitations. First, the follow‐up time in the cohort was 4.69 years, which was relatively short to identify new cancer cases. An evaluation of weaker associations, for example, between albumin levels and gastric cancer risk was precluded due to low case numbers. Besides, analyses by cancer types and subgroups were also restricted. Second, it is known that blood biochemistry markers would undergo successive changes individually over time. However, with limited follow‐up duration, the albumin concentration used in this analysis was at one single time point, and changes of cancer risk along with albumin concentrations were not analyzed. Third, most participants enrolled in this study were baseline healthy subjects, and the albumin concentrations mainly ranged between 35 and 50 g/L. Hypoproteinemia was unable to be analyzed separately with only 0.20% (167/82061) of albumin concentrations below 35 g/L. Fourth, since the proportion of females in this study was less than 25%, these results were limited when interpreting associations for females. Last but not the least, though our study added to evidence that albumin was significantly associated with total cancer risk as well as risks of lung, colorectal, and liver cancer, the biological plausibility was still moderate and the clinical significance remains to be elucidated. The positive results with serum albumin were novel and should be interpreted cautiously.

On the other hand, this study had strengths over previous studies. This analysis was based on a population‐based cohort with well‐organized follow‐up. Adjustment for potential confounders was comprehensively performed including adjustment for C‐reactive protein, which helped when exploring weak associations. It is noteworthy that this study first conducted RCS analysis to analyze the nonlinear relationship between pre‐diagnostic serum albumin and cancer risk.

In conclusion, our findings suggest that pre‐diagnostic serum albumin is inversely and linearly associated with cancer risk among the Chinese population. And the significant association between albumin and overall cancer risk is mainly attributed to lung, colorectal and especially, liver cancer. This study provides evidence that albumin may be valuable to the prediction and stratification of cancer risk in the general population. Since albumin concentration is widely used in physical examination, this marker is easy to obtain and well accepted by the population. When combined with other detection indexes, it becomes an indicator of both feasibility and considerable economic value before the invasive examination. Nevertheless, the biological mechanism and clinical significance remain to be elucidated. Population studies with longer follow‐up time as well as experimental studies are further required.

## AUTHOR CONTRIBUTIONS

Ni Li, Min Dai, Jie He, and Shouling Wu were responsible for the study concepts and design. Zhuoyu Yang, Yadi Zheng, Zheng Wu, Yan Wen, Gang Wang, and Shuohua Chen contributed to data acquisition. Fengwei Tan and Jiang Li were involved in quality control and data interpretation. Zhuoyu Yang, Yadi Zheng, Zheng Wu, and Yan Wen were responsible for paper preparation. Statistical analyses were performed by Zhuoyu Yang, Yadi Zheng, Zheng Wu, and Yan Wen. Ni Li and Jie He contributed to manuscript editing. All authors approved the final version of the manuscript.

## ETHICS APPROVAL AND CONSENT TO PARTICIPATE

The studies involving human participants were reviewed and approved by the ethics committee of the Kailuan General Hospital. The patients/participants provided their written informed consent to participate in this study. This study was performed in accordance with the Declaration of Helsinki.

## CONFLICT OF INTEREST

The authors declare no potential conflict of interest.

## Data Availability

The datasets for this manuscript are not publicly available under the regulation of both the National Cancer Center of China and the Kailuan Group. However, data are available from the authors upon reasonable request. Requests to access the datasets should be directed to JH, hejie@cicams.ac.cn and SLW, drwusl@163.com.
